# Impairment of Wnt/β-catenin signaling in blood cells of patients with severe cavitary pulmonary tuberculosis

**DOI:** 10.1371/journal.pone.0172549

**Published:** 2017-03-23

**Authors:** Lin Fan, Hongbo Shen, Huichang Huang, Rui Yang, Lan Yao

**Affiliations:** 1 Clinic and Research Center of Tuberculosis, Shanghai Key Lab of Tuberculosis, Shanghai Pulmonary Hospital, Tongji University School of Medicine, Shanghai, China; 2 Unit of Anti-tuberculosis Immunity, Key Laboratory of Molecular Virology and Immunology, Institute Pasteur of Shanghai, Chinese Academy of Sciences, Shanghai, China; The University of Hong Kong, HONG KONG

## Abstract

Tuberculosis (TB) remains as a leading infectious disease worldwide. Our previous study showed interferon (IFN)-γ and CD3 T cell impairment in patients with severe cavitary pulmonary TB (PTB). However, the cause of the change in immune responses during the progression of TB is still poorly understood. In this study, eight newly diagnosed patients with severe cavitary and mild lesion non-cavity PTB were recruited, and three healthy volunteers were recruited as the control. RNA extracted from blood was tested by whole genome oligo microarrays. A PCR array was used to further test the same samples. Two additional groups of patients were recruited according to the same criteria with healthy control(HC) recruited as well and subjected to peripheral blood mononuclear cell isolation (PBMC)and analysis of TCF-7, β-catenin, cyclin D2, IFN-γ, and tumor necrosis factor (TNF)-α expression in CD14- cells (lymphocytes) and CD14+ cells by quantitative PCR. The changes of expression of β-catenin, CD69+ and IFN-γ by CD3+, CD14- and CD14+ cells in vitro with stimulation of LiCl were tested by flow cytometry. Whole genome oligo microarrays showed a significant decrease in expression of the Wnt signaling pathway in severe PTB patients. Further analysis of the Wnt pathway by PCR array indicated that TCF-7, β-catenin, and cyclin D2 expression was significantly reduced in severe PTB patients compared with mild PTB patients. In the additionally recruited patients, TCF-7, β-catenin, and cyclin D2 were expressed in both CD14+ and CD14- cells, while β-catenin was decreased significantly in CD14- cells compared with CD14+ cells in severe PTB patients, and IFN-γ and TNF-α expression in CD14- cells was also reduced significantly in severe PTB patients. β-catenin can directly trigger T cell activation and IFN-γsecretion in PBMCs stimulated for 24 hours. These findings indicate that Wnt pathway and its key genes, such as β-catenin, were impaired in blood cells of patients with severe PTB. Therefore, Wnt/β-catenin pathway is closely associated with T cell proliferation and TB lesion deterioration.

## Introduction

Tuberculosis (TB) remains as a serious infectious disease worldwide. There were an estimated 9.6 million TB cases globally, and TB killed 1.5 million people in 2014 based on the updated global TB report [1]. Controlling TB is a significant challenge in countries or areas with high TB burden. It is essential to clarify the immunological regulation during the development of TB in the host to further elucidate targets for therapeutic vaccines.

TB cases and tuberculin skin test-positive healthy donors have variant immune responses to MTB (*Mycobacterium tuberculosis*) antigens [2–3]. A large variation has been observed in immune responses of patients with differing severities of active pulmonary tuberculosis (PTB). We speculated that a critical pathway or molecules might be involved in the change of TB from mild to severe stages. Our previous study [3] showed obvious decreases in MTB antigen-specific interferon (IFN)-γ and CD3+ T cells in the blood of patients with severe cavitary TB compared with mild TB patients. Therefore, the impairment of INF-γ responses when TB lesions become severe needs clarification.

In the present study, we determined the main differences in gene expression between severe and mild PTB patients, and further analyzed the reduction of IFN-γ expression during the deterioration of PTB.

## Materials and methods

### Study population

Patients were recruited at Units Three and Five in the Department of Tuberculosis, Shanghai Pulmonary Hospital between January 1, 2015 and May 30, 2016. Patients were included in the study according to the following criteria: (1) diagnosed as newly treated active PTB confirmed by bacteriology with all information regarding bacteriology or pathology, and typical radiological manifestation; (2) patients with severe cavitary PTB with at least one large cavity of ≥3 cm in diameter or at least three cavities regardless of the diameter of cavities or patients with mild lesion PTB without a cavity (patients had mild lesions in ≤two lung fields and no cavity) as reported previously [3], a cavity, or non-cavitary lesions in lungs, which could be observed in a chest computed tomography (CT) scan; (3) patients had never been treated for TB or the anti-TB treatment duration was within 1 week; (4) subjects provided informed consent for the study. During the same period, healthy volunteers were recruited for the control group. All procedures were performed in accordance with the protocol approved by the Ethics Committee of Shanghai Pulmonary Hospital. Written informed consent was obtained from all participants who agreed to participate in this study. Patients with an immunosuppressive status or had received immunosuppressive agents or anti-tuberculosis chemotherapy for more than 1 week were excluded from the study.

All information regarding the characteristics of thoracic radiology (cavity diameter, number and location of cavities, symptoms, and complications) was recorded carefully.

### Peripheral blood mononuclear cell (PBMC) isolation

PBMCs were isolated from EDTA-treated blood by density gradient centrifugation using Ficoll-Paque Plus (GE healthcare, UK) and then cultured in RPMI 1640 medium supplemented with 10% fetal bovine serum, 2 mM glutamine, and 50 U/ml penicillin, and 50 μg/ml (Invitrogen, Carlsbad, CA).

### Expression analysis by whole genome oligo microarray

Venous blood samples (10 ml) were collected in heparin tubes from eight TB patients (four cases of severe cavitary PTB and four cases of mild lesion PTB) and three healthy volunteers. Total RNA from each blood sample was harvested using TRIzol (Invitrogen). Briefly, homogenized samples were incubated for 5 minutes at 15 at 30°C to permit complete dissociation of nucleoprotein complexes. Add 0.2 ml of chloroform per 1 ml of TRIzol Reagent. Shake the tubes vigorously and incubate them at 15–30°C for 2–3 min before centrifugation at 12,000 × *g* for 15 min at 4°C. RNA remains in the aqueous phase. Transfer the aqueous phase to a fresh tube. Precipitate the RNA by mixing with isopropyl alcohol. Wash the RNA pellet with 75% ethanol. Dissolve air-dried RNA in RNase-free water. RNA was further purified with an RNasey Mini Kit (product number 74104; Qiagen).

RNA quantity and quality were measured using a NanoDrop ND-1000 spectrophotometer. Denaturing gel electrophoresis was conducted to assess the RNA integrity. Total RNA from each sample was amplified and transcribed into fluorescent cRNA using Agilent's Quick Amp Labeling protocol, One-Color (product number 5190–0442; Agilent Technologies, Santa Clara, CA). The labeled cRNA from each sample was hybridized onto a Whole Genome Oligo Microarray slide (4×44 K, Agilent Technologies) with Agilent SureHyb hybridization chambers. After hybridization using the Agilent Gene Expression Hybridization Kit (product number 5188–5242; Agilent Technologies) and washing with Gene Expression Wash Buffer (product numbers 5188–5325 and 5188–5326; Agilent Technologies), the processed slides were scanned with an Agilent microarray scanner (product number G2565BA; Agilent Technologies) using the settings recommended by Agilent Technologies.

Microarray data were extracted using Agilent Feature Extraction software. Datasets were normalized in GeneSpring GX using the Agilent FE one-color scenario (mainly median normalization).

### Microarray data analysis

Gene expression ratios of the normalized signal intensity of severe cavitary PTB samples to the normalized signal intensity of mild lesion PTB samples were calculated from the microarray data. To identify differentially expressed genes between the two groups, we performed fold-change filtering and paired Student’s t-tests. The cutoff value was a 2-fold change. Genes with expression levels that differed by at least 5-fold from the mean in at least one sample were selected for further evaluation. The significance threshold was fold changes (ratios) of ≥2.0 or ≤0.5 and P-values of ≤0.05. Only differentially expressed genes were chosen for further investigation.

Gene Ontology (GO) categorization and pathway analysis were carried out using GO database (http://www.geneontology.org/) terms and Kyoto Encyclopedia of Genes and Genomes (KEGG) pathways (http://www.genome.jp/kegg/).

### PCR array analysis of the Wnt signaling pathway

The Human Wnt Signaling Pathway RT^2^ Profiler PCR Array profiles the expression of 84 genes related to Wnt-mediated signal transduction. This array was selected for measurement of Wnt-related gene expression based on the results of gene and pathway analyses to validate the microarray data. The expression of these genes had a high fold change in gene analysis and were identified by a high factor in differently expressed KEGG pathways. Total RNA from an additional 11 samples (four cases of severe cavitary PTB, four cases with no cavity PTB, and three healthy volunteers) was prepared for quantitative PCR (qPCR) in the same manner as for the microarray analysis. Purified RNA was reverse transcribed into cDNA using Superscript III RT (Invitrogen, Karlsruhe, Germany). Quantification of gene expression was performed by qPCR. GAPDH and ACTB were used as endogenous controls.

### CD14+ and CD14- cell isolation

Twenty-two patients satisfied the inclusion criteria were recruited into this study. Fresh PBMCs from these patients were used to isolate CD14+ and CD14- cells using CD14 MicroBeads (Miltenyi Biotec, Berfisch Gladbach, Germany) according to the manufacturer’s instructions. Briefly, PBMCs were magnetically labeled with microbeads conjugated with monoclonal mouse anti-human CD14 antibodies and separated on a column placed in the magnetic field. The magnetically labeled CD14+ cells were retained in the column and eluted as positively selected CD14+ cells. The unlabeled cells were collected as CD14- cells.

### qPCR for quantification of gene expression

Total RNA was extracted from freshly isolated CD14+ and CD14- cells using RNA column enrichment procedures according to the manufacturer’s protocol (Zymo Research, CA). Total RNA were reverse transcribed into cDNA with a random/oligo dT primer mixture using a kit from Vazyme Biotech (Nanjing, China). The cDNA was added to 2× Syber Green Mastermix (Toyobo Life Science, Shanghai, China) to amplify target genes in triplicate reactions for each gene. Negative cDNA controls (no cDNA) were prepared in parallel with each run. Primer sequences for TCF-7, β-catenin, cyclin D2, IFN-γ, tumor necrosis factor (TNF)-α, and β-actin are listed in Table 1.

**Table 1:** Primers used for qPCR.

Gene	Forward primer	Reverse primer	Product size
**TCF-7**	5’-CTGACCTCTCTGGCTTCTACTC-3’	5’-CAGAACCTAGCATCAAGGATGGG-3’	99bp
**β-catenin**	5’-CACAAGCAGAGTGCTGAAGGTG-3’	5’-GATTCCTGAGAGTCCAAAGACAG-3’	146bp
**CyclinD2**	5’-GAGAAGCTGTCTCTGATCCGCA-3’	5’-CTTCCAGTTGCGATCATCGACG-3’	104bp
**IFN-γ**	5’-GCAGGTCATTCAGATGTAGCGG-3’	5’-TGTCTTCCTTGATGGTCTCCACAAC-3’	181bp
**TNF-α**	5’-CTCTTCTGCCTGCTGCATTTG-3’	5’-ATGGGCTACAGGCTTGTCACTC-3’	135bp
**β-actin**	5’-GCCCTGAGGCACTCTTCCA-3’	5’-TGTTGGCGTACAGGTCTTTGC-3’	120bp

qPCR was performed in triplicate in an ABI PRISM 7900HT Sequence Detection System using the following conditions: 95°C for 1 min, and then 40 cycles of 95°C for 15 s and 60°C for 60 s. A melting curve for each primer ensured amplification of a single product. The housekeeping gene β-actin was used as internal control for normalization.

### Activation of WNT pathway tested by flow cytometry

PBMCs isolated from TB patients were cultured in at 37°C in a humidified atmosphere with 5% CO2, and stimulated with Lithium chloride (LiCl, Sigma) at 20mM for 24 hours. Then, cells were collected and stained with Pacific blue-conjugated anti-human CD3 (Clone SP34–2, BD), FITC-conjugated anti-human CD14 (Clone HCD14, Bioelgend), BV605-conjugated anti-human CD69 (clone FN50, Biolegend), BV711-conjugated anti-human IFN—γ (Clone 4S.B3, Biolegend), APC-conjugated anti-human betta-catenin (Clone 196624, R&D). After staining, cells were fixed with 2% formaldehyde-PBS (Protocol Formalin, Kalamazoo, MI) and subjected to run on a BD LSRF ortessa flow cytometer (BD Biosciences, Qume Drive, San Jose, CA). Lymphocytes were gated based on forward- and side-scatters, and at least 40,000 gated events were analyzed using Summit Data Acquisition and Analysis Software (Dako Cytomation).

### Statistical analyses

Differences between two groups were assessed by the unpaired, two tailed Student’s t-test using Prism 5.01 software. Values of p < 0.05 were considered statistically significant.

## Results

### Patient population

We first recruited 11 participants whose whole venous blood samples were applied to whole genome oligo microarrays. Eight patients had active PTB and three participants were healthy volunteers. Four of the eight patients had severe cavitary PTB, and four of them had mild lesion PTB. The average age was 34 ± 13.3 years. Severe PTB, mild PTB, and healthy volunteers had evenly distributed ages and sexes and no complications. After performing whole genome oligo microarray and data analyses, we recruited 22 more patients with active PTB using the same inclusion criteria and 8 healthy volunteers as healthy controls. Eleven of the 22 patients had mild lesion PTB, and 11 of them had severe PTB. The average age was 38.3 ± 16.6 years. Two severe PTB cases were accompanied by tuberculous meningitis, two PTB cases had tuberculous pleurisy, and one severe PTB case had intestinal TB.

### Wnt pathway expression is impaired in severe cavitary PTB

**Fig 1:**
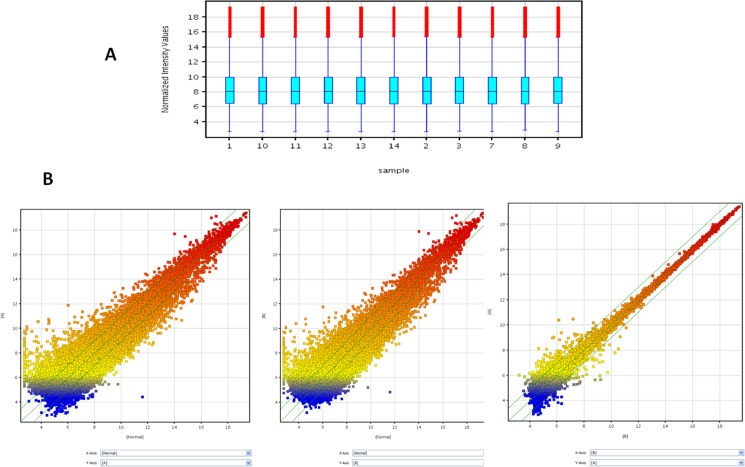
Expression analysis using whole genome oligo microarrays: Distribution of datasets of 11 blood samples. After normalization, the distributions of log2 ratios among the samples were nearly the same (A); Scatter plot analysis used to visualize the expression variation between A (severe PTB group) and normal (*Left*), or B (mild lesion PTB group) and normal (*middle*), and A and B (*right*). X and Y axes are the normalized signal values of each sample (log2 scaled). Green lines are fold changes (fold change value was 2.0) (B).

**Fig 2:**
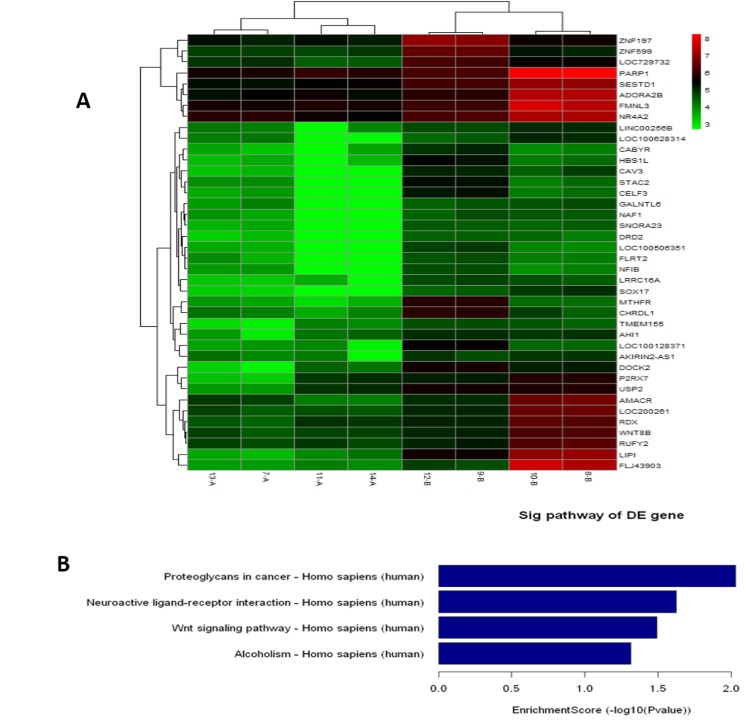
Heat map and hierarchical clustering. Normalized expression levels of 40 genes downregulated in group A compared with group B were used in the cluster analysis. Red indicates high relative expression, and green indicates low relative expression. SOX17 and WNT8B are genes in the Wnt signaling pathway(A); Bar plot showing the top ten enrichment scores [-log10 (P-value)] of significant pathway enrichment (B).

**Fig 3:**
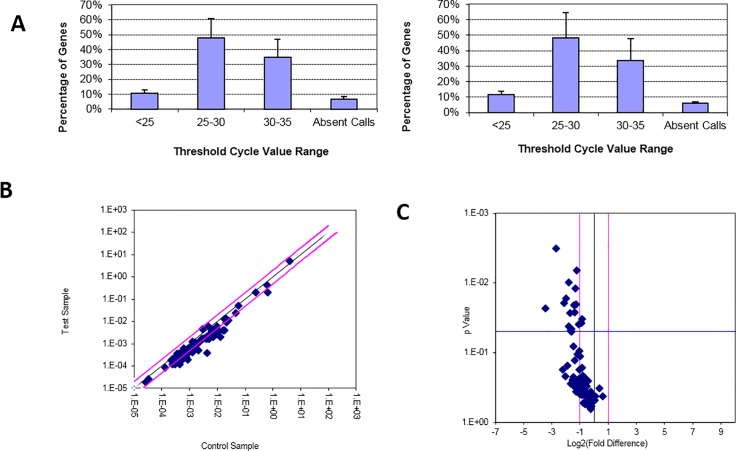
Expression profiles of Wnt pathway-related genes: Threshold cycle value range of A (left) and B (right) samples in the Human WNT signaling pathway PCR array (A); Black line indicates fold changes [(2 ^ (-ΔCt)] of 1. Pink lines indicate the desired fold change in the gene expression threshold (B); Black line indicates a fold change in gene expression of 1. Pink lines indicate the desired fold change in the gene expression threshold. Blue line indicates the desired threshold for the p-value of the t-test (C)

**Fig 4:**
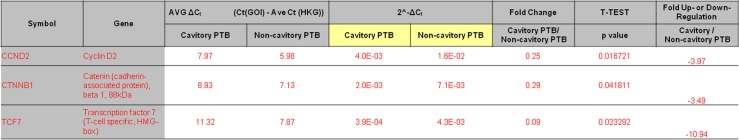
Expression of Wnt pathway related genes of cyclin D2, β-catenin, and TCF-7 using the Wnt Signaling Pathway RT^2^ Profiler PCR Array.

To dissect immune differences between severe cavity PTB and mild lesion non-cavitary PTB patients, we analyzed gene expression in blood samples from patients with severe cavitary or mild lesion PTB and healthy controls by microarray (see S1 Table, the GEO accession number was GSE95563). We normalized raw microarray data and filtered low intensity data to screen for differentially expressed genes. After normalization, the box plot (Fig 1A) showed that the distributions of log2 ratios among the samples were nearly the same, indicating high quality microarray data. Next, we assessed the expression variation among samples in the three groups. The scatter plot showed many differently expressed genes among these samples (Fig 1B). To identify differentially expressed genes with statistical significance, we performed volcano plot filtering between any two groups (fold change: ≥2.0; p-value: ≤0.05) (Fig 1B). Through hierarchical clustering, we found similar differences between severe PTB patients versus healthy controls and mild PTB patients versus healthy controls, such as the mitogen-activated protein kinase signaling pathway and cell adhesion molecules. However, we found distinguishable gene expression profiles between samples from severe PTB patients versus mild PTB patients (Fig 2A). Next, we carried out pathway analysis of differentially expressed genes based on the latest KEGG database. Four biological pathways were identified with significant enrichment of differentially expressed genes (the p-value cutoff was 0.05) (Fig 2B). The Wnt signaling pathway was one of them (Fig 2B, Table 2). To further compare the expression of Wnt pathway-related genes in samples from severe cavitary and mild lesion non-cavitary PTB patients, we used a human Wnt signaling pathway PCR array that profiles the expression of 84 genes related to Wnt-mediated signal transduction. Distributions of threshold cycle values showed that the data qualities were reliable (Fig 3). Our results revealed significant decreases in the expression of some genes in severe cavitary PTB samples compared with those in mild lesion non-cavitary PTB samples, especially β-catenin, cyclin D2, and TCF-7 (Fig 4). These results imply a decrease in Wnt pathway expression in severe cavity PTB patients.

**Table 2:** Differences in pathway expression of severe cavitary PTB patients compared with mild lesion PTB patients.

PathwayID	Definition	Fisher-Pvalue	Enrichment_Score	Genes
**hsa05205**	Proteoglycans in cancer—Homo sapiens (human)	0.009309794	2.03106	CAV3//RDX//WNT8B
**hsa04080**	Neuroactive ligand-receptor interaction—Homo sapiens (human)	0.02375825	1.624186	ADORA2B//DRD2//P2RX7
**hsa04310**	Wnt signaling pathway—Homo sapiens (human)	0.03233289	1.490355	SOX17//WNT8B
**hsa05034**	Alcoholism—Homo sapiens (human)	0.048711	1.312373	ADORA2B//DRD2

### Expression of β-catenin decreases in CD14- cells of severe cavitary PTB patients

**Fig 5:**
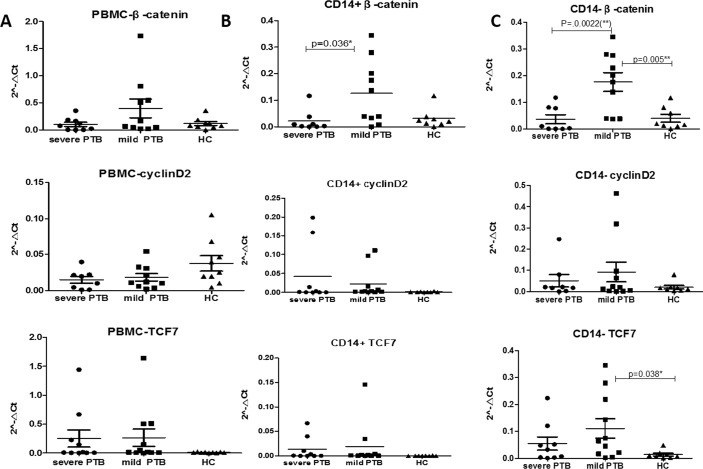
qPCR analyses of cyclinD2, β-catenin, and TCF-7 in PBMCs (A), CD14+ cells (B), and CD14- (C) cells.

To compare the expression of Wnt pathway-related genes in cell subpopulations between severe cavitary and mild lesion non-cavitary PTB patients, we detected the expression of β-catenin, cyclin D2, and TCF-7 in PBMCs, CD14+ cells (monocytes) and, CD14- cells (lymphocytes). We found that expression of β-catenin was lower in samples from severe cavity PTB patients compared with those from mild lesion non-cavitary PTB patients, especially in CD14+ and CD14- cells (Fig 5A–5C). β-Catenin was differently expressed in CD14- cells. Moreover, expression of β-catenin decreased more significantly in CD14- cells (Fig 5C, p = 0.0022) than in CD14+ cells (Fig 5B, p = 0.036). Expression of cyclin D2 and TCF-7 was also lower in CD14- cells, but they were expressed similarly in CD14+ cells between severe cavity PTB and mild lesion non-cavitary PTB patients. Therefore, we presumed that canonical Wnt pathway involving β-catenin was impaired in cavitary PTB, especially in CD14- cells.

### Expression of Th1 cytokines decreases in CD14- cells of severe cavitary PTB patients

**Fig 6:**
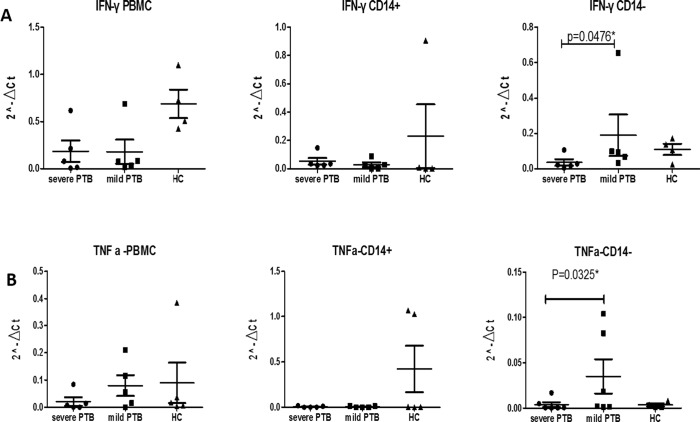
qPCR analysis of major Th1 cytokines TNF-α (A) and IFN-γ (B) in PBMCs, CD14+, and CD14- cells from blood samples of patients with severe or mild lesion PTB.

To verify the functional differences of CD14- cells in patients with severe cavity PTB and non-cavitary PTB, we measured the expression of IFN-γ and TNF-α by qPCR. We found that expression of IFN-γ in CD14- cell of severe cavity PTB patients was significantly lower than that in mild lesion non-cavitary PTB patients (Fig 6A, p = 0.0476), but there was no obvious difference between PBMCs and CD14+ cells. Similar results were obtained for TNF-α (Fig 6B, p = 0.0325). These data indicated that expression of Th1 cytokines decreased in CD14- cells of severe cavitary PTB patients compared with to mild lesion non-cavity PTB patients in accordance with the decreased expression of Wnt pathway-related genes.

### β-catenin can directly trigger T cell activation and IFN-γ secretion in PBMC of PTB

**Fig 7:**
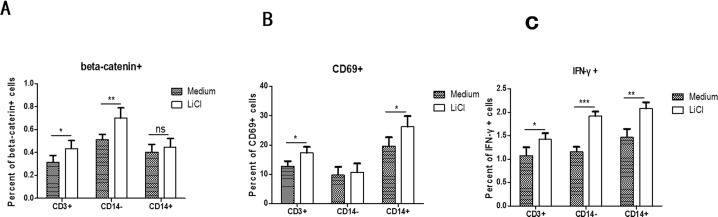
Cell staining results tested by flow cytometry, the percentage of β -catenin +cells (A) by CD3+, CD14-,CD14+ cells using LiCl stimulation or medium, the percentage of CD69+ cells(B) by CD3+, CD14-, CD14+ cells using LiCl stimulation or medium, the percentage of IFN-γ(C)by CD3+,CD14-,CD14+ cells using LiCl stimulation or medium. * P〈0.05, ** P〈0.01.

To verify β-catenin, one of the Wnt signaling molecule can directly activate the T cells and its function, we used LiCl which stimulates Wnt pathway stimulating PBMC 24 hours in vitro from PTB patients, tested and compared the changes of expression of β-catenin, CD69 and IFN-γby flow cytometry, we found that expression of β-catenin by CD3+ and CD14- cells, IFN-γby CD3+ CD14+ and CD14- cells, percent of CD69+ CD3+ and CD69+CD14+ cells increased after LiCl stimulation. This result indicated that β-catenin can directly influence T cells activation and Th1 secretion in PTB. See Fig 7A–7C)

## Discussion

Based on microarray data analysis and screening, our results showed that Wnt pathway expression decreases significantly with simultaneous downregulation of critical genes related to Wnt-mediated signal transduction in blood cells of patients with severe cavitary PTB. These results indicate that expression of the Wnt pathway might be impaired when TB becomes severe.

The Wnt signaling pathway is evolutionarily conserved and has critical roles in the development of many organs. The Wnt1 gene, originally named Int-1, was identified in 1982, which was activated by integration of mouse mammary tumor virus [4]. Dysregulated expression of Wnt signaling is involved in the initiation of various tumors such as hematological malignancies and gastric, lung, pancreatic and breast tumors [5–9]. In addition to the variety of tumors, Wnt proteins are linked to many other human diseases [10].

The role of the Wnt signaling pathway is becoming more defined in immunity. The Wnt pathway controls the proliferation of progenitor cells and affects cell decisions [11–13]. Wnts are secreted lipid-modified glycoproteins that bind to Frizzled (Fzd) receptors and activate multiple signaling pathways including canonical and non-canonical pathways. The former regulates transcription of target genes through the β-catenin/TCF pathway, whereas the non-canonical pathway is independent of β-catenin. Upregulation of Wnt signaling in hematopoietic stem cells (HSCs) increases expression of Notch1 genes, indicating that Wnt and Notch pathways might cooperate to maintain HSC homeostasis [14]. Several reports demonstrate that exposure to Wnts can condition dendritic cells (DCs) to a regulatory state, while β-catenin in DCs drives T regulatory cell differentiation via the Toll-like receptor 2 and plays a critical role in tolerance and autoimmune disorders [15–17]. The Wnt pathway also regulates CD4+/CD8+ differentiation and B cell development [18–20].

Studies of the Wnt signaling pathway associated with TB pathogenesis have been reported in recent years. However, its regulatory mechanism and role in TB pathogenesis have been poorly understood. One study [21] showed that two single nucleotide polymorphisms in the Wnt pathway are associated with TB risk. In addition, the genetic variant in the Wnt signaling pathway might be associated with susceptibility to TB in the Chinese population. Several studies indicate Wnt pathway expression in macrophages and DCs. In an aerosol experiment of mice infected with MTB [22], Fzd1 expression was increased on macrophages in response to MTB, and its ligand, Wnt3a, reduced TNF release from MTB-infected macrophages. Wn5a is induced in human macrophages in response to mycobacteria, and Wnt3a-mediated anti-inflammatory effects on mycobacteria-infected macrophages occur via the Wnt/β-catenin signaling pathway [23]. Wnt6 is expressed in granulomatous lesions of MTB-infected mice and involved in the proliferation and differentiation of macrophages [24]. Canonical Wnt signaling promotes macrophage apoptosis in response to *Mycobacterium bovis Bacillus Calmette-Guerin* (BCG) infection, and Wnt/β-catenin signaling reduces BCG-induced macrophage necrosis through a reactive oxygen species-mediated Poly (ADP-ribose) polymerase/AIF-dependent pathway [25]. These studies imply that the Wnt canonical pathway plays an advantageous role in anti-TB immune regulation of macrophages in the host.

Our previous study showed that *M*. *tuberculosis* antigen-specific IFN-γ and CD3+CD4+ T cells decrease significantly in the blood of patients with severe cavitary PTB. Based on the link between the decreased number of T cells and downregulation of the Wnt pathway in the microarray analysis of severe PTB patients, we speculated that downregulation of the Wnt pathway might be involved in the impairment of CD3+ T cell proliferation and activation during TB lesion deterioration. PCR array analysis of the Wnt signaling pathway verified significant reductions in the expression of critical genes in the canonical Wnt pathway, such as β-catenin, cyclin D2, and TCF-7. We recruited an additional 22 patients with the same inclusion criteria and confirmed the downregulation of β-catenin and Th1 cytokines in CD14- cells (lymphocytes) compared with CD14+ cells. Based on comprehensive analysis of our data, we propose the following possible conclusions: (1) Key genes in the Wnt pathway are expressed in both lymphocytes and macrophages or monocytes in TB patients. (2) Expression of Wnt signaling is significantly impaired in lymphocytes compared with monocytes from blood of patients with severe cavitary PTB. (3) IFN-γ and TNF-α expression is much lower in lymphocytes than in monocytes from the blood of patients with severe cavitary PTB.

Our study first demonstrated that the Wnt pathway might be involved in the deterioration of human TB. Stimulation of Wnt/β-catenin pathway can directly activate T cells and its secretion of Th1 cytokines in PBMC of PTB. Combined with our previous studies, we showed that the Wnt pathway should be closely linked to decreased CD3+ T cells and impaired IFN-γ in severe TB, and the critical proteins of the Wnt pathway, β-catenin might participate in regulating and maintaining the proliferation and differentiation of T cells in TB pathogenesis. When TB lesions deteriorate in the severe stage, Wnt protein on T cells is impaired and further affects the activity and number of CD3+ T cells, thereby impairing secretion of Th1 cytokines. Another study in mice demonstrated that activation of Wnt signaling in T cell lineages causes spontaneous T cell activation and severe T cell lymphopenia [26]. The underlying regulatory mechanism of the Wnt pathway in T cells of TB patients and the association of other pathways simultaneously regulating T cells and Th1 cytokine secretion have been unclear. There may be important immunotherapeutic targets in the Wnt pathway expressed in T cells. These speculations regarding the regulatory mechanism of the Wnt pathway during the progression of TB will be studied further.

Among the 22 PTB patients, we observed several interesting cases: one patient with severe PTB accompanied by intestinal TB had very low expression of β-catenin, TCF-7, and cyclin D2. This patient died at 1 month after anti-TB chemotherapy. Several mild lesion PTB patients with high expression of β-catenin recovered and their pulmonary lesions were absorbed smoothly during treatment, whereas some other patients with lower expression of β-catenin showed slow absorption of pulmonary lesions in the follow-up chest CT scanning. Therefore, the underlying findings of this study include the following. (1) Some critical proteins of the Wnt pathway might be predictive factors of the outcome of TB treatment. (2) Some proteins of the Wnt pathway might be biomarkers to distinguish severe and mild TB (there is still the lack of a sensitive biomarker to define TB lesion severity except image features and some uncertain markers [27–28]). (3) The Wnt pathway or unknown molecule(s) associated with the Wnt pathway might be a potential immunotherapeutic target for TB patients. From the comparison of pathway between severe and mild PTB group, ADORA2B was also in the list which has role in the inflammation, however, its role might not be associated with impaired T cells proliferation in the progress of TB. Therefore, in this study we focus on the study of wnt pathway.

In summary, we found impairment of the canonical Wnt pathway in blood cells of patients with severe cavitary PTB. β-Catenin, the key protein of the Wnt pathway, showed a simultaneous decrease in expression. The Wnt pathway was expressed in both lymphocytes and macrophages and showed obvious impairment of expression in CD14- cells compared with CD14+ cells in blood of severe TB patients. Th1 cytokines were downregulated in CD14- cells of severe cavitary TB patients. Wnt/β-catenin pathway should be the element closely associated with T cell activation and TB lesion deterioration.

## Supporting information

S1 Table Gene expression profiling data from eight patients with PTB and three healthy controls. (XLS)
